# Supplementary material for: Arbitrary order exceptional point induced by photonic spin–orbit interaction in coupled resonators
Source: Nat Commun. 2019 Feb 19;10:832. doi: 10.1038/s41467-019-08826-6 (PMC6381179; doi:10.1038/s41467-019-08826-6)
Supplement: Supplementary file 1 — Supplementary Information [file 41467_2019_8826_MOESM1_ESM.pdf]

Supplementary Information

Arbitrary order exceptional point induced by photonic spin-orbit interaction in coupled resonators

Wang *et al*.

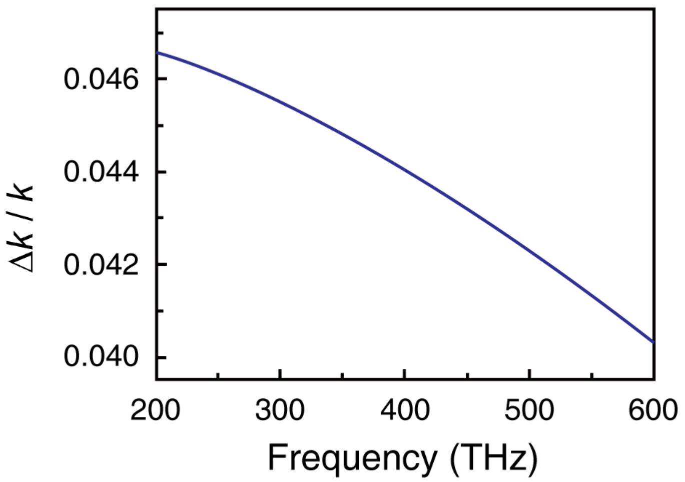

Supplementary Figure 1 Variation of plasmon wavevector as a function of frequency for the lossy waveguide. k denotes the wavevector without loss and Δk denotes the difference of the real part of the wavevector before and after introducing the loss into the waveguide. We have set $\gamma_{\mathrm{wg}} = 0.3$. As $\Delta k / k$ is on the order of 10^{-2}, the contribution of γ_{wg} to the phase of the coupling parameters can be neglected.

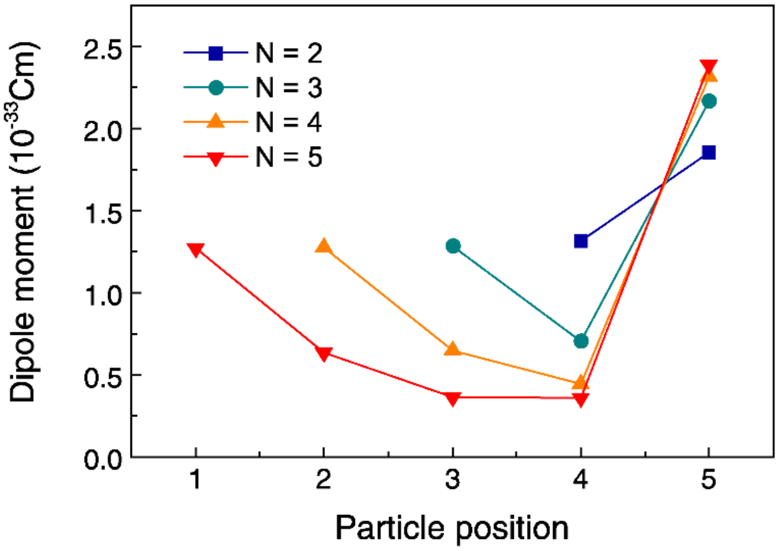

Supplementary Figure 2 Higher order exceptional point for enhancing dipole emission. Resonance electric dipole moment of each sphere under left-handed circular polarisation excitation for *N* spheres. Energy from the incident light is collected by the left spheres and transferred to the 5th sphere which has a larger dipole moment. The spacing of the 1-4th spheres is $d = \lambda_{plas}$ and the spacing between the 4th and the 5th spheres is $d = 1.5\lambda_{plas}$. With such a spacing arrangement, the guided wave coming from each of spheres 1-4 gives a constructive interference at the 5th sphere and hence an enhancement of dipole moment at that site. Note that the dipole moment at the last sphere increases as the number of spheres in the array increases.

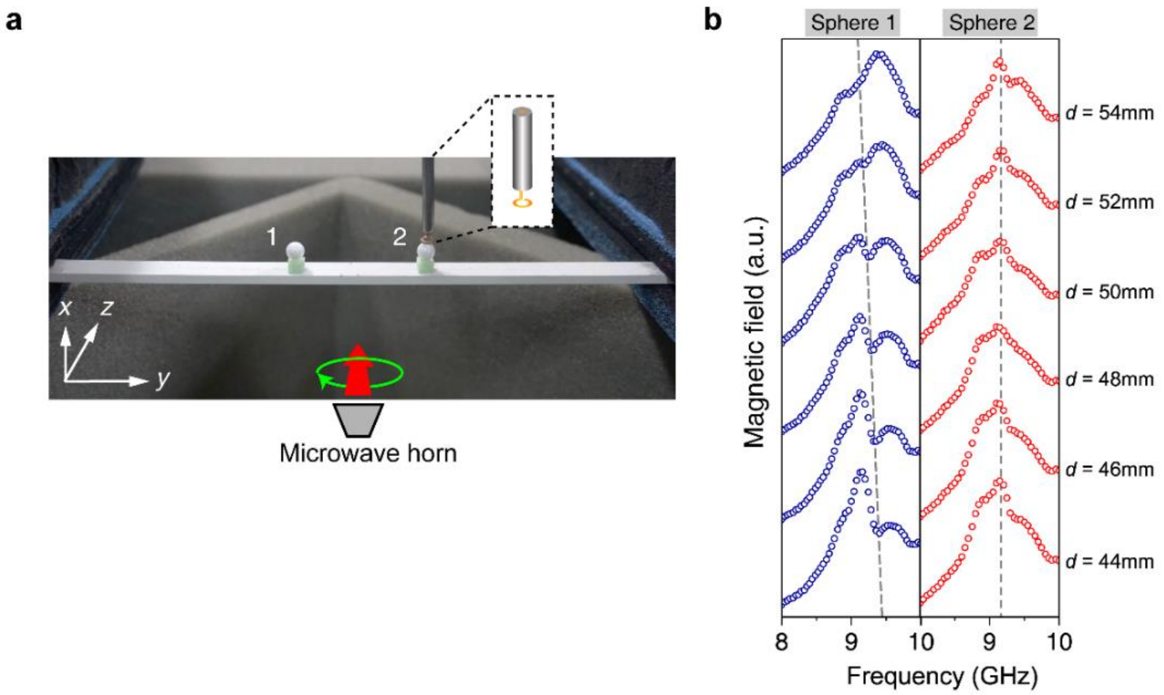

Supplementary Figure 3 Measured magnetic dipole responses under right-handed circular polarisation excitation. a Microwave experimental setup. Two identical dielectric spheres were placed on a dielectric strip waveguide with the gap distance controlled by a foam spacer. The spheres have diameters of 5.56 mm. A probe measured the magnetic field near the spheres under circular polarisation excitation. **b** Measured magnitude of the magnetic field on top of the two spheres. The dashed lines mark the positions of the local minima (Sphere 1) and the maxima (Sphere 2) of the field.

a

b

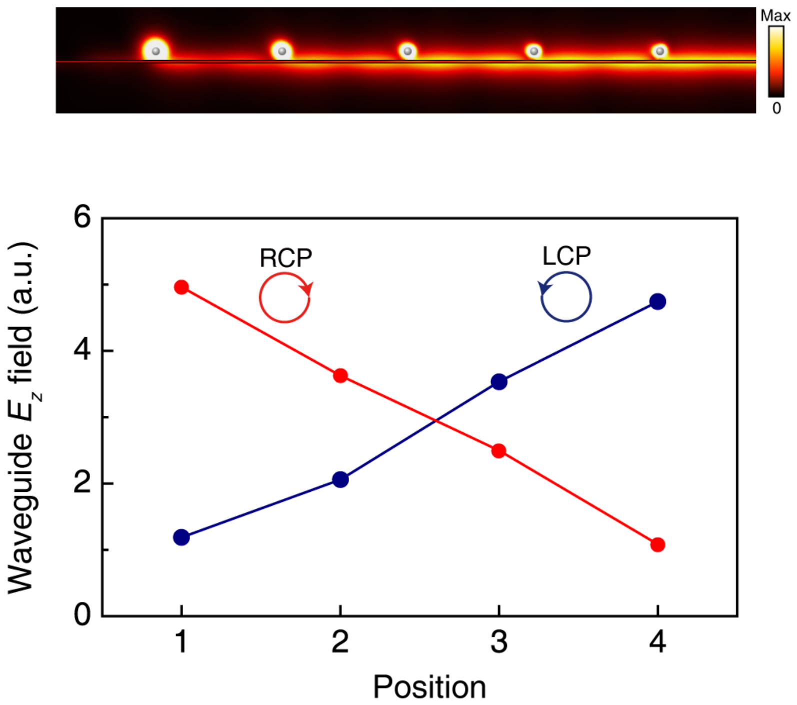

Supplementary Figure 4 Position dependence of the waveguide field. a Numerical simulation result of the electric field magnitude for the model system of Fig. 3a under left-handed circular polarisation excitation. **b** Measured E_z field magnitude near the lower surface the waveguide (right below the spheres) for the microwave system of Fig. 6. Under left-handed (right-handed) circular polarisation excitation, the field increases from left (right) to right (left).

Supplementary Note 1
Monotonic behavior of induced dipole moments at higher-order exceptional points

Here we present an analytical derivation for the behaviors of chiral dipole moments at the EPs. Consider the case of LCP, the rate equation for N coupled particles can be expressed as:

$$\frac{da_i}{dt} = -\mathrm{i}\omega_0 a_i - \frac{\Gamma}{2} a_i - \mathrm{i}\kappa_{ij} \sum_{j=1}^{i\text{-}1} a_j - \sqrt{\gamma_\mathrm{c}}\, a_\mathrm{in}, \tag{1}$$

Take the N = 3 case as an example, substitute the harmonic wave expressions $a_\mathrm{in} = A_\mathrm{in} e^{-\mathrm{i}\omega t}$ and $a_i = A_i e^{-\mathrm{i}\omega t}$ into the above equation, we obtain

$$(\omega - \tilde{\omega}_0) A_1 + \mathrm{i}\sqrt{\gamma_\mathrm{c}} A_\mathrm{in} = 0, \tag{2}$$

$$(\omega - \tilde{\omega}_0) A_2 - \kappa_{21} A_1 + \mathrm{i}\sqrt{\gamma_\mathrm{c}} A_\mathrm{in} = 0, \tag{3}$$

$$(\omega - \tilde{\omega}_0) A_3 - \kappa_{32} A_2 - \kappa_{31} A_1 + \mathrm{i}\sqrt{\gamma_\mathrm{c}} A_\mathrm{in} = 0, \tag{4}$$

where $\tilde{\omega}_0 = \omega_0 - \mathrm{i}\Gamma/2$. The static solution to the above equations is

$$A_1 = \frac{-\mathrm{i}\sqrt{\gamma_\mathrm{c}} A_\mathrm{in}}{\omega - \tilde{\omega}_0}, \tag{5}$$

$$A_2 = \frac{-\mathrm{i}\sqrt{\gamma_\mathrm{c}} A_\mathrm{in}}{(\omega - \tilde{\omega}_0)^2} (\omega - \tilde{\omega}_0 + \kappa_{21}), \tag{6}$$

$$A_3 = \frac{-\mathrm{i}\sqrt{\gamma_\mathrm{c}} A_\mathrm{in}}{(\omega - \tilde{\omega}_0)^3} \left[(\omega - \tilde{\omega}_0)^2 + (\omega - \tilde{\omega}_0)(\kappa_{32} + \kappa_{31}) + \kappa_{32}\kappa_{21} \right]. \tag{7}$$

At $\omega = \omega_0$, we have $\omega - \tilde{\omega}_0 = \mathrm{i}\Gamma/2$. Assume the particle array has a period of $d = \lambda_\mathrm{plas}$ as in Fig. 3a, we have $\kappa_{ij} = \kappa_{21} = -\mathrm{i}\kappa_{21}^0, \forall i > j$, and the electric dipole moments can be expressed as

$$p_1 = \left| \frac{2\sqrt{\gamma_\mathrm{c}} A_\mathrm{in}}{\Gamma} \right|,\ p_2 = \left| \frac{2\sqrt{\gamma_\mathrm{c}} A_\mathrm{in}}{\Gamma} \left(\frac{\Gamma - 2\kappa_{21}^0}{\Gamma} \right) \right|,\ p_3 = \left| \frac{2\sqrt{\gamma_\mathrm{c}} A_\mathrm{in}}{\Gamma} \left(\frac{\Gamma - 2\kappa_{21}^0}{\Gamma} \right)^2 \right|. \tag{8}$$

Similar derivations can be done for arbitrary number of spheres, in which case the electric dipole moments are

$$p_i = \left| \frac{2\sqrt{\gamma_\mathrm{c}} A_\mathrm{in}}{\Gamma} \left(\frac{\Gamma - 2\kappa_{21}^0}{\Gamma} \right)^{i-1} \right|. \tag{9}$$

Both κ_{12}^0 and Γ take positive real values and $|(\Gamma - 2\kappa_{21}^0)/\Gamma| < 1$ according to the results in Fig. 2l, so we have $p_i < p_{i-1}$, which indicates a monotonic decreasing trend of the dipole moments.
